# A Shorter Route to Antibody Binders *via* Quantitative *in vitro* Bead-Display Screening and Consensus Analysis

**DOI:** 10.1038/srep36391

**Published:** 2016-11-07

**Authors:** Sylwia A. Mankowska, Pietro Gatti-Lafranconi, Matthieu Chodorge, Sudharsan Sridharan, Ralph R. Minter, Florian Hollfelder

**Affiliations:** 1 Department of Biochemistry, University of Cambridge, 80 Tennis Court Road, Cambridge CB2 1GA, UK; 2 Antibody Discovery and Protein Engineering, MedImmune Ltd, Milstein Building, Granta Park, Cambridge, CB21 6GH, UK

## Abstract

Affinity panning of large libraries is a powerful tool to identify protein binders. However, panning rounds are followed by the tedious re-screening of the clones obtained to evaluate binders precisely. In a first application of Bead Surface Display (BeSD) we show successful *in vitro* affinity selections based on flow cytometric analysis that allows fine quantitative discrimination between binders. Subsequent consensus analysis of the resulting sequences enables identification of clones that bind tighter than those arising directly from the experimental selection output. This is demonstrated by evolution of an anti-Fas receptor single-chain variable fragment (scFv) that was improved 98-fold *vs* the parental clone. Four rounds of quantitative screening by fluorescence-activated cell sorting of an error-prone library based on fine discrimination between binders in BeSD were followed by analysis of 200 full-length output sequences that suggested a new consensus design with a *K*_d_ ∼140 pM. This approach shortens the time and effort to obtain high affinity reagents and its cell-free nature transcends limitations inherent in previous *in vivo* display systems.

High-affinity protein binders with defined specificity have become increasingly important reagents in basic research, large-scale proteomic studies, and also represent the fastest-growing segment of the pharmaceutical market^1^. A variety of display systems^2^ has emerged for the directed evolution^3^ of binding proteins, providing direct access to recombinant binding reagents^4^,^5^. In addition to the widely used phage display^6^,^7^,^8^,^9^,^10^, cell-free formats^11^, such as ribosome^12^,^13^,^14^, mRNA^15^,^16^, mHaeIII^17^,^18^, CIS^19^ or SNAP display^20^,^21^,^22^,^23^,^24^, exist that remove *in vivo* constraints from the selection process and sample larger diversity space. In these display systems the protein of interest (POI) is fused to its coding DNA (or RNA). This linkage allows identification of a displayed protein that interacts with an immobilised target after selection from a large library, typically containing >10^9^ protein variants. Such ‘affinity panning’ selections are largely based on off-rates (*k*_off_) and highly dependent on the conditions employed, i.e., the duration and number of washes in the panning procedure as well as the antigen concentration. Variants are recovered, if their *K*_d_ falls below a threshold, which is not precisely defined. The threshold is a function of the experimental protocol and confounding factors, in particular stabilisation of protein interactions through avid binding and variations in growth rates between variants during the *in vivo* amplifications. In systems where a greater number of proteins can be displayed (e.g., ~10^4^ copies on bacteria^25^,^26^,^27^,^28^,^29^ or 30,000 copies on yeast^30^) selections can be made on the basis of the number of bound, fluorescently-labeled target molecules. For every single library member this proxy for a binding curve is measured by flow cytometry that ranks and sorts binders. The replacement of the ‘panning’ step by a more quantitative, direct readout of occupancy with the binding partner should provide a more faithful reflection of the binding constant (*K*_d_) and reduce the need for cumbersome biophysical characterizations of hundreds, if not thousands, of individually prepared variants after the selection step.

An *in vitro* equivalent to the multivalent natural display systems was recently introduced, consisting of a megavalent variation of SNAP display (dubbed BeSD, Bead Surface Display^31^). Here up to a million copies of DNA and protein are assembled on a bead in a multi-step procedure (Fig. 1) that involves *in vitro* compartmentalisation in water-in-oil emulsion droplets. In this method, a stable genotype-phenotype link is created by fusing the protein of interest to a SNAP-tag, which binds covalently to benzylguanine (BG) labeled DNA^31^. As in cell display, BeSD has the potential to rank libraries of up to 10^5^ protein variants and carry out selections, by fluorescence-activated cell sorting in under an hour. Previously BeSD had only been used to display the small peptide hemagglutinin (HA)-tag (size: 1.3 kDa for the HA and 19 kDa for the fusion partner, SNAP-tag)^31^. To expand the scope of BeSD to selection of single-chain variable antibody fragment (scFv) binders, the folding efficiency during the *in vitro* expression had to be improved to functionally display the much larger SNAP-scFv-HA fusion (resulting in a 47 kDa protein construct). Here we use BeSD^31^ for directed evolution of an scFv against Fas receptor (FasR, CD95), which belongs to the tumour necrosis factor receptor (TNFR) superfamily that contains valuable drug targets (e.g., TRAIL-R1^32^,^33^,^34^ and TRAIL-R2^35^).

As a starting point for an affinity-maturation campaign, we chose the anti-Fas receptor scFv E09, which was originally selected from a naïve antibody phage library^36^,^37^. The svFV E09 has previously been affinity matured by six rounds of ribosome display (RD)^36^,^37^, enabling us to make comparisons to the current evolution method. Four FACS-based screening rounds together with a phylogenetic analysis of the selected scFv mutants yielded two scFv variants with up to two orders of magnitude improved binding affinity for FasR.

## Results

### Bead Surface Display of functional antibody fragments with different affinities

The anti-FasR scFv E09 was displayed on the bead surface as a SNAP-scFv-HA fusion *via* DNA spiking anchors (see Supplementary Fig. 1). Binding assays were performed *in vitro* on-bead to assess antigen binding by flow cytometry as a proxy for efficient expression and correct folding (Fig. 2). The assay utilised the antibodies as fluorescent probes, and resulted in significant increases in median fluorescence signal (MFS) being observed for the display of SNAP-scFv-HA, detected with an anti-HA antibody, or an anti-Fc antibody, which detects Fc-fused FasR bound to the scFv (Fig. 2A). A similar fluorescence signal was observed for display of SNAP-HA and SNAP-scFv-HA (Fig. 2A), suggesting that that the expression of an scFv fusion is as efficient as that of the HA-tag and reaches the maximally achievable display level (~10^6^ per bead^31^). The fluorescence distributions of the negative controls (without the BG on the bead) varied between the two detection modes, suggesting that there is a fraction of dysfunctional, misfolded scFv present attached non-specifically to the bead surface itself, which can be detected with the anti-HA antibody. However, these scFv molecules do not bind to the target (and consequently are not detected with the anti-Fc antibody). It has been shown that certain scFv antibodies can be prone to aggregation as a result of misfolding^38^,^39^, and this could explain the observed background signal during the on-bead display measurement. Nevertheless, the presence of a negligible fraction of misfolded scFv on the bead was not expected to interfere with the performance of the binding assay during the selection process. In order to achieve uniform surface display levels on beads for the SNAP-scFv-HA and the SNAP-HA fusion proteins, prior optimisation of *in vitro* expression and display efficiency was necessary. In particular, the time and temperature of the incubation step had to be re-evaluated (see Supplementary Fig. 2 and Supplementary Protocol 1).

In order to establish sufficiently stringent conditions to select for high affinity scFv in library screenings, the resolution of the display system was investigated. We compared the flow cytometric on-bead binding signals of the anti-FasR variants with a range of affinities: Ep6b_B01, E09, E09_Y58S (with the *K*_d_ values of 0.18 nM, 8.6 nM and 187 nM, respectively)^37^ and a non-binder CEA6 scFv^40^. Each scFv was displayed on the bead surface and subsequently incubated with 1 nM, 10 nM and 30 nM of the antibody-labelled FasR-Fc (Fig. 2B). This experiment demonstrated that the median fluorescence signal corresponds to the affinity of the scFv and the increase in the signal indicates tighter binding. The difference in the MFS between weakest (187 nM) and strongest binders (0.18 nM) increased from 1.8-fold to 8.4-fold when the antigen concentration was dropped from 30 nM to 1 nM. This apparent correlation supported the idea^41^ that — in a monovalent selection system — a reduced antigen concentration increases the selection stringency, such that, at equilibrium, a greater proportion of displayed higher affinity antibodies will carry the fluorescent antigen compared to lower affinity antibodies. These observations suggest that BeSD is capable of discriminating between variants on the basis of binding affinity, provided that an appropriate antigen concentration is chosen.

### Screening of an scFv library generated by error-prone PCR

**Figure 1:**
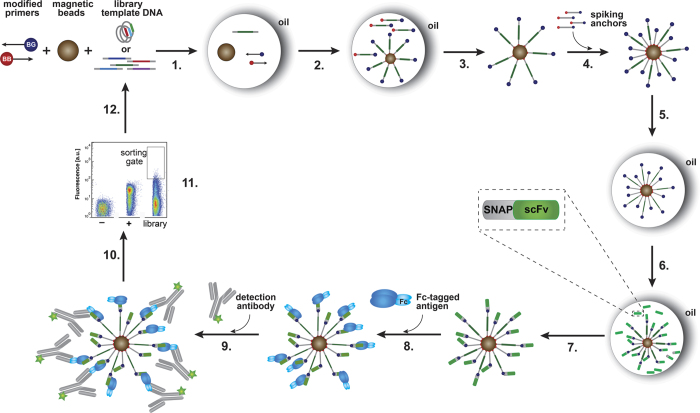
Schematic overview of a selection round using Bead Surface Display (BeSD). **(1)** The DNA template, a streptavidin-coated magnetic bead, benzyl guanine (BG)-conjugated primers and biotin (BB)-conjugated primers are encapsulated in water-in-oil droplets, in such a way that the Poisson distribution dictates that there is no more than one DNA template per bead; **(2)** DNA is amplified by emulsion PCR (ePCR) to give >10^6^ copies, of which ~100–1,000 copies are captured on-bead; **(3)** the droplet contents are de-emulsified and the beads are washed; **(4)** BG-BB DNA anchors are added (as additional valencies for display); **(5)** compartmentalisation of single beads together with IVTT (*in vitro* transcription/translation) mix in water-in-oil droplets; **(6)** protein is expressed from the bead-immobilised templates (4 hour expression at 25 °C); **(7)** de-emulsification liberates beads that are now displaying the protein of interest (e.g., SNAP-scFv-HA), followed by washes to remove the IVTT mixture and unbound excess of expressed protein; **(8)** incubation with the target (FasR-Fc) followed by washes to remove unbound target; **(9)** incubation with secondary antibody (anti-Fc DyLight^®^488); **(10)** washes to remove excess detection antibody; **(11)** beads that bind (and show fluorescence above a chosen threshold) are sorted by flow cytometry (FACS) at a rate of ~10^6^ per hour; **(12)** recovery of the DNA that encoded clones identified as binders. The Figure was adapted from Diamante *et al*.^31^ which is available under the terms of the Creative Commons Attribution License (http://creativecommons.org/licenses/by/3.0/).

**Figure 2:**
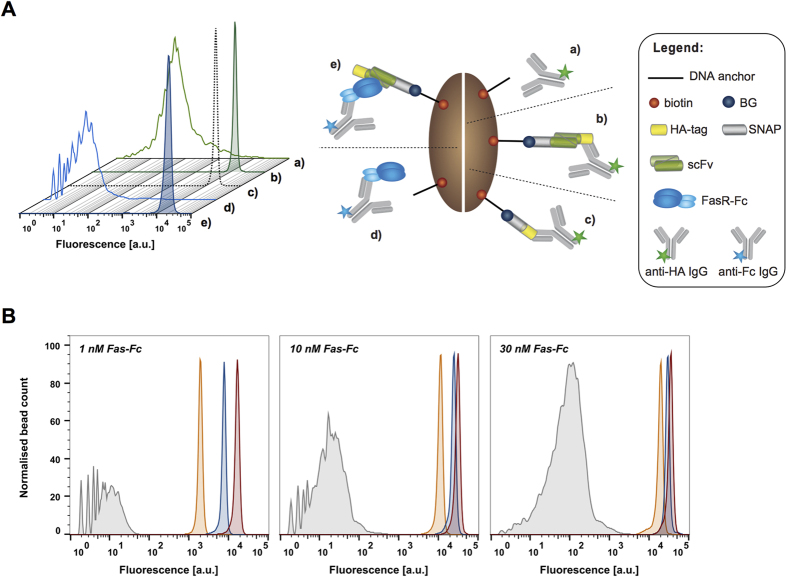
On-bead assays and affinity measurement. (**A**) The SNAP-E09 scFv-HA construct was expressed *in vitro* in the presence of streptavidin-coated beads coupled to spiking anchors without the BG moiety (negative controls; histograms a and d) or to spiking anchors conjugated to the BG (histograms b and e). The beads were then subjected to on-bead display (cartoons a and b) and binding assays (cartoons d and e). In the on-bead display assay the SNAP-scFv-HA was detected with Alexa488-labelled anti-HA antibody (histograms a and b; green). Fluorescence distribution from the flow cytometric analysis showed a 250-fold increase in the median fluorescence signal compared to the fluorescence of beads without bound ligands. The SNAP-scFv-HA showed a similar fluorescence signal (histogram b; filled green) to the SNAP-HA construct displayed on beads (histogram c; black dotted line), suggesting that maximum on-bead display was reached. For the on-bead binding assay, the same SNAP-scFv-HA construct was detected with a fluorescently labelled anti-Fc antibody (that detects the binding of the FasR at 1 nM; histograms (d and e;) blue), showing >1,250-fold signal increase over the background. (**B**) On-bead binding assays for different scFv variants analysed by flow cytometry: a non-binder (CEA6, grey) and three binders with increasing affinity, namely E09_Y58S (*K*_d_ ~180 nM, orange), E09 (*K*_d_ ~9 nM, blue) and EP6b_B01 (*K*_d_ ~0.2 nM, red). Beads displaying the respective scFv variants were incubated in the presence of 1 nM, 10 nM and 30 nM of the target FasR-Fc (detected with a fluorescently labelled anti-Fc antibody). The peak of the fluorescence distribution obtained by flow cytometry correlates to the affinity of the displayed scFv and shows a clear difference between the variants. The resolution of scFv binders is improved at lower antigen concentration, since, at equilibrium, a greater proportion of higher affinity antibodies than lower affinity antibodies is able to bind to the available antigen.

**Figure 3:**
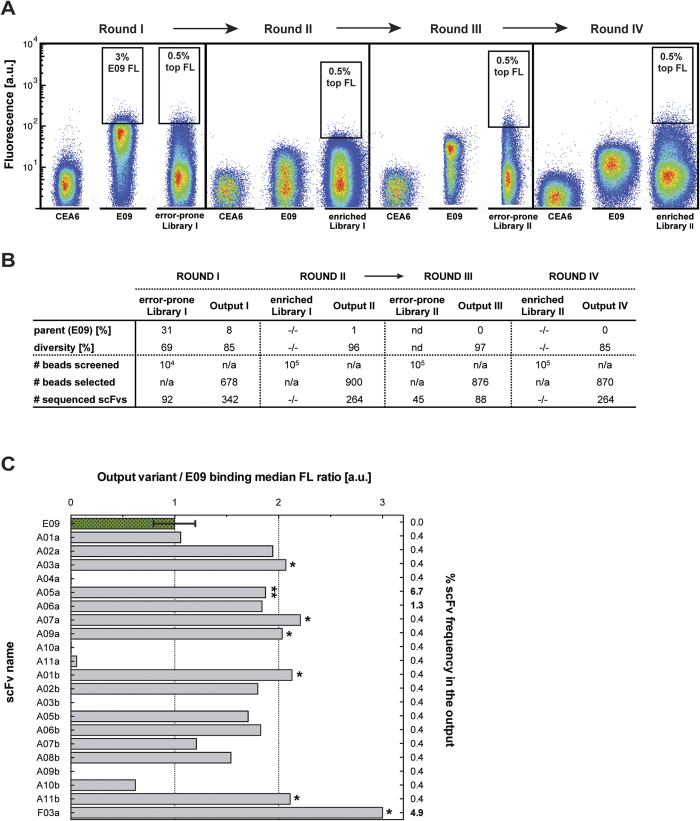
Overview of the scFv selections carried out by BeSD. **(A)** Four selection rounds were carried out and the fluorescence distributions obtained by flow cytometry in each selection round are presented as density plots. In addition to the actual library in each round the non-binder (CEA6) and parent (E09) and were screened as controls (all in the presence of 1 nM FasR-Fc). The position of the sorting gate is indicated by a black box and the percentage of the sorted population falling into the gate is shown. **(B)** Summary of the selection campaign. The error-prone randomisation of the second selection round output is indicated with an arrow. Each output had high diversity of scFv amino acid sequence (with a decreasing proportion of parent E09 in each subsequent round). **(C)** 21 randomly selected scFvs from the fourth output were tested in on-bead binding assays. The plot shows the binding ratio of each variant over the E09 scFv (green bar, triplicate measurements), and the frequency of each variant in the selection output (numbers on right hand side). Seven scFvs either showing the largest improvements in binding (signal >2-fold higher than E09; marked with ‘*’) or appearing with the highest frequency in the output (marked with ‘**’) were expressed in *E. coli* and analysed biophysically (Table 1). Only one clone, F03a, failed in bacterial expression, and thus was not characterised.

Beginning with the scFv E09 (parent)^37^ as a DNA template, an error-prone library with a low mutation rate of ~1.7 amino acid changes per scFv (Library I) was created. Selection cycles were performed using an improved protocol (Fig. 1) based on that of Diamante *et al*.^31^ (as discussed above), in which 1–2 × 10^5^ library members were screened in each round. To find an optimal threshold concentration for selection of the improved scFvs, the equilibrium binding titration curves were determined (Supplementary Fig. 3). The apparent *K*_d_ value of the parent was 4.3 nM (±0.3), thus the library screening was done at an antigen concentration of 1 nM. It is worth noting that the on-bead *K*_d_ measurement was highly reproducible; the standard deviation between the individual normalised values was only 13% and between the derived *K*_d_ values 25% (Supplementary Fig. 4). By comparison, the variability of *K*_d_ values in yeast display was reported to be 30%^42^. This screening procedure (pictured in Fig. 1) was repeated until a total of four rounds of selection had been performed (see Fig. 3A) with intermittent re-randomisation and an increasingly narrow sorting gate. In the first selection round (round I) a permissive sorting gate was set (i.e. on 3% of the parent fluorescent population and the same gate was used for selecting variants from the Library I), followed by more stringent rounds with the sorting gate set on the top 0.5% library members (rounds II-IV). To further explore diversity of the scFv variants, another round of mutagenesis by error-prone PCR was performed to introduce mutations after round II (made using the same mutation rate as before, resulting in Library II), now starting from the pool of scFv variants selected in rounds I and II.

It is noteworthy that the selections were done entirely *in vitro* — the recovered scFv sequence from each round was PCR-assembled into the full SNAP-scFv-HA construct using a high fidelity polymerase (see Methods). Subsets of each output were also cloned for the sequence analysis, which demonstrated highly diverse variant sequences (Fig. 3B). The frequency of wild-type E09 occurrence decreased continuously in successive selection rounds, indicating that the enrichment of improved variants at the expense of the starting species was successful (Fig. 3B).

### Characterisation of the selected scFvs

After the fourth selection round the output was cloned into the pISNEX *in vitro* expression vector (Supplementary Fig. 9) or into the vector pCantab6 (for periplasmic bacterial expression)^43^. 21 randomly selected clones were tested in on-bead binding assays to identify binders of improved affinity (Fig. 3C). Additionally, the proportion and affinity improvement of the output binders from our selection was compared to the output of the previous ribosome display campaign (that underwent six rounds of evolution^36^ rather than four as in this work). 88 clones from the round IV output of BeSD were picked and expressed in *E. coli* and the supernatants were tested in a binding plate assay as described by Chodorge *et al*.^36^. The supernatant screen showed that hit rate in both outputs was comparable: the improved variants (defined as mutants with a binding signal three times above the parent) constituted 45% of the ribosome display output (data not published) and 35% for the BeSD output (Supplementary Fig. 5). Moreover, the on-bead assay (performed with 21 variants, Fig. 3C) showed that 44% of the tested variants demonstrated higher binding signal when compared to the parent scFv. This result agreed with the plate based supernatant screen and it emphasises that the on-bead approach faithfully reflects conventional biophysical measurements.

Six clones (A01b, A03a, A07a, A09a, A11b, F03a), out of the 21 tested in the on-bead binding assay, with the highest affinity improvements (>2-fold increase of MFS in on-bead binding assay over E09, see Fig. 3C) and the most abundant variant (A05a, contributing ~7% of the output of round IV) were expressed in *E. coli* and their binding kinetics were tested by bio-layer interferometry (BLI) (with the exception of F03a, which failed in bacterial expression). The parent scFv E09 and EP6b_B01, a high affinity variant from ribosome display^37^, were analysed alongside the selection outputs as references, and Table 1 summarises the *K*_d_, *k*_on_ and *k*_off_ values obtained. The binding constants of the scFv antibodies showed that all variants selected by BeSD had improved, low nM affinities. The largest gain was demonstrated by the variant A07a, being ~19-fold over the parent and ~2-fold over the EP6b_B01 clone. As the BLI was insufficiently sensitive to discriminate slow *k*_off_ values, the scFv A07a was converted to an IgG to exclude avidity effects in the determination of *K*_d_ values, by virtue of performing the analysis with a low density IgG-coated chip surface and using surface plasmon resonance (SPR). In this format A07a, from BeSD, and EP6b_B01, from ribosome display, were 47–fold and 228–fold improved, respectively.

### Analysis of the sequence alignment of the output reveals affinity-relevant hotspots

**Figure 4:**
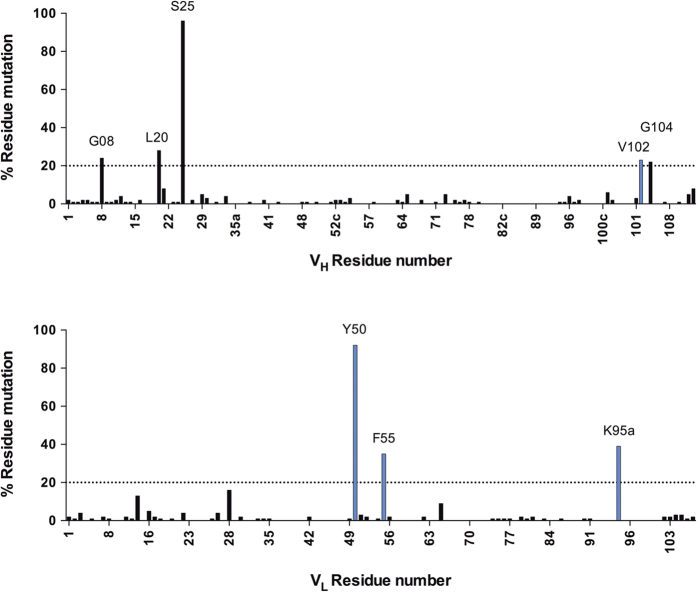
Hotspot identification based on amino acid sequence analysis of the fourth round selection output. The percentage of residue mutated (when compared to the parent, E09, sequence) in the fourth round selection outputs (223 sequences analysed), and positions that were mutated in >20% of all sequences were qualified as hotspots. The mutated residues falling in CDR regions are highlighted in blue, and framework regions in black.

Panning selections have the drawback that they typically yield a proportion of proteins with weak affinity that are still sufficient to pass a threshold defined by the antigen concentration and washing steps, which demands screening hundreds, if not thousands, of individually prepared variants in biophysical assays to provide additional characterisation. In BeSD (and in other multivalent display systems, e.g., yeast display) the readout is quantitative, i.e., it reports directly on ligand occupancy and thus more directly reflects *K*_d_. We therefore probed whether the quantitative readout from BeSD could define a binding consensus, simply based on sequencing rather than time-consuming biophysical analysis of individual clones. To examine this hypothesis, 223 randomly selected clones form the fourth selection round output were sequenced (by Sanger sequencing) and aligned to the parent E09 scFv. The resulting alignment revealed 8 hotspots^36^, defined as positions mutated in greater than 20% of the sequences analysed (Fig. 4). The mutations in those positions were progressively enriched in the course of selections (Supplementary Fig. 6). In the hotspot positions each residue was preferably changed to a specific amino acid (contributing to more than 80% of all mutations occurring in that position, data not shown), suggesting that those favoured mutations were specifically selected during the scFv evolution, as they are beneficial for its biophysical properties. The residue V_L_ Y50 (numbered according to the Kabat system^44^) was the only position that did not have a clearly dominating mutation, and the tyrosine was mutated either to serine or histidine (covering 50% or 48% of mutations occurring in this position, respectively). This observation of two alternative residues in position V_L_ Y50 raised the possibility of deleterious negative epistatic interactions^45^,^46^,^47^ between these residues and other mutations and suggested a another possible driving force behind selection of either histidine or serine in this position. This bifurcation in the evolutionary history of the final variants^48^ would be indicative of a rugged fitness landscape^49^,^50^.

### Phylogenetic analysis of the scFv from the final selection output allows building a consensus scFv binder with greatly improved affinity

**Figure 5:**
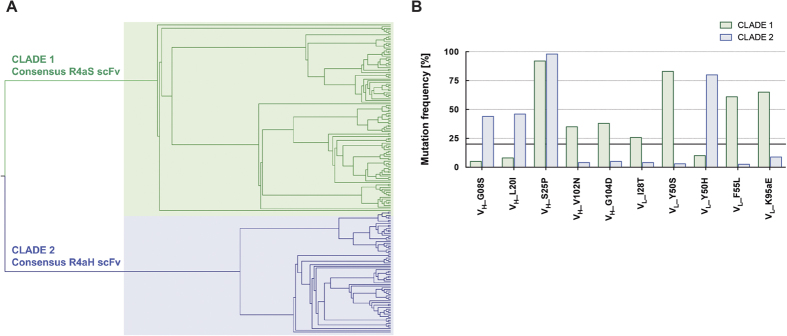
Phylogenetic analysis of the fourth selection round output suggests two consensus designs for R4aS and R4aH. **(A)** 193 full-length amino acid sequences from the fourth selection round output scFvs were aligned using the MUSCLE algorithm (online), then the phylogenetic tree was created with CIPRES and visualised in FigTree. The two distinct clades were observed, suggesting that 2 separate lineages of scFvs emerged in the course of the scFv evolution. **(B)** The graph presents the dominant mutations in the hotspot positions (mutations present in >20% of sequences) in the Clades 1 and 2. The mutations contributing to more than 20% of all analysed sequences in the corresponding clade were classified as *consensus* mutations. The co-existence of these mutations suggests that no negative epistatic interactions occur between them. Additionally, the fact that they were greatly enriched in the last selection round indicates that they were necessary to improve the target binding properties of the scFvs. The dominant residues for each subsequent clade were assembled into R4aS and R4aH scFv consensus mutants. Interestingly, the scFv containing all consensus mutations from Clade 2 was also identified as the A05a scFv - the most abundant scFv variant in the output of the fourth round of BeSD selection (see Fig. 3C and Table 1), and has ~3-fold improved *K*_d_ value (compared to the parent E09 scFv). The second consensus scFv, R4aS, was created (by introducing the relevant mutations to E09 backbone by site-directed mutagenesis), and subsequently its affinity was measured by BLI (see Table 1).

To investigate the relationships between the hotspot mutations a phylogenetic analysis was performed. To uncover potential epistatic interactions between the hotspot mutations, the unique sequences of the fourth selection round output (193 sequences) were aligned using the MUSCLE algorithm^51^ (online) and the resulting phylogenetic tree (displayed in Fig. 5) was created with CIPRES^52^. The mutations found in more than 20% of the sequences in each clade were classified as consensus mutations for that clade. In addition to the hotspot positions (defined above), residue I28 was mutated to a tyrosine in 26% of sequences from the Clade 1, qualifying it also as a consensus mutation. To verify the contribution of each of the identified consensus mutations to the scFv’s binding capability, the individual mutations were introduced into the parent and tested by BLI (Supplementary Fig. 7). Mutations V_H_ S25P and V_L_ Y50S each resulted in a 4-fold increase in the affinity for the FasR (compared to the *K*_d_ of the parent scFv E09). Consequently, to evaluate the epistatic interactions between the mutations, the two scFv consensus mutants (named R4aS and R4aH) were constructed (Fig. 5). The scFv R4aH containing all consensus mutations from Clade 2 had already been identified in our experiments as the A05a scFv, the most abundant scFv variant in the output of the fourth round of BeSD selection (see Fig. 3C and Table 1), and showed 3-fold improvement in the *K*_d_ value compared to the parent E09. The second consensus scFv, R4aS, was created by introducing the mutations from the Clade 1 into the E09 backbone by site-directed mutagenesis. R4aS was expressed both as scFv and IgG and its affinity determined by BLI and SPR (Table 1). The R4aS variant resulted in a high affinity IgG with a *K*_d_ of 0.14 nM and a *K*_d_ gain over parent of 98-fold (measured by SPR), representing tighter binding than the best experimentally selected variant, A07a (*K*_d_ ∼0.29 nM; Table 1). When modelled on the structure of the E09 scFv in complex with the FasR derived by Chodorge *et al*.^37^ (Supplementary Fig. 8), neither the individual mutations nor the consensus scFv were predicted to improve the binding, because they are remote from the interaction surface.

## Discussion

This work has established a new powerful methodology in which the evolution of protein binders is achieved by a combination of quantitative *in vitro* screening and subsequent analysis of consensus mutations. The combination of these two approaches identifies binders with affinities that surpass those of the experimental output:

(i) *Quantitative in vitro screening*. First, experimental *in vitro* selections with a quantitative readout that reports on affinity were carried out based on a new bead display system, BeSD, which is used here for the first time for evolution of an antibody fragment. The selection of the 290 pM binder A07a and an improvement of 47-fold after four rounds of BeSD was possible from a moderately sized (10^5^-membered) error-prone library (in contrast to a 49-fold improvement obtained after 6 rounds of ribosome display selections and extensive subsequent screening of hundreds of individually prepared variants, as reported before with a 10^7^-fold larger library)^37^. The high quality single clone characterisation in BeSD leads to a high positive rate (confirmed by biophysical characterisation by BLI and SPR), because the titration-like FACS binding assays provide a much more quantitative way of assessing binding strength than affinity panning (i.e. selection against an immobilised target, where stringent control of selection threshold is hard to achieve). The benefit of a quantitative selection system is to improve the selection efficiency: the majority of hits were true positives with improved *K*_d_ and satisfying the selection threshold. Strikingly, only seven variants needed to be individually prepared and screened to isolate A07a from the BeSD output. By contrast, typical panning outputs in phage or ribosome display selections need to undergo the laborious step of re-screening hundreds, if not thousands, of individually prepared variants in microplate assays in order to distinguish those variants genuinely selected for improvements in the desired function from those which simply persist in the selection process. In short, as binders with similar affinity result in both approaches, the much larger library diversity screened in ribosome display seems to be compensated by the better quality of the screen in BeSD, reporting directly on *K*_d_ (while involving dramatically fewer experiments).

A further advantage of BeSD is that the *in vitro* approach transcends *in vivo* host constraints, allowing more precise control over the protein folding environment (e.g., control of redox conditions and the presence of particular molecular chaperones) allow simplified selections for protein stability (e.g., thermostablility, which typically results in the death of the host cell^53^) and also obviates the need for cumbersome transformations of variant libraries into a host cell. The new methodology goes beyond other bead-based platforms on record^54^,^55^ as none of the reported platforms have demonstrated the ability to display antibody fragments or quantitatively discriminate binders of varying affinities.

(ii) *Consensus analysis*. The quantitative readout of BeSD provides information that actively guided the design of consensus mutants. Highly enriched mutations were identified, and by applying a phylogenic analysis, a consensus scFv variant was assembled. The phylogenetic grouping of consensus patterns provides further guidance to avoid unproductive interactions between residues, based on genotypic incompatibility (i.e., negative epistatic interactions^46^,^49^). This treatment led to creation of antibody R4aS with a 98-fold higher affinity than the parent. The high quality of selections is the precondition that such an approach can be successful, as reported in a hotspot analysis by Boder^56^ that was also able to characterise binding for each single clone quantitatively. Nowadays, the availability of the high throughput sequencing technologies enhances the depth and breadth of insight into the course of directed evolution of proteins^57^,^58^. Deep sequencing analysis has identified nanomolar binders from naïve selections^59^, but with short reads epistatic interactions are likely to be obscured. Also, limited sequencing length^60^, confined the readout to the CDR^59^, while in this work beneficial mutations were identified across the entire gene. Also the limited sequencing length in prior work confined the readout to the CDR loops, while in this work beneficial mutations were identified across the entire gene. Although in this work the sequencing depth is moderate (200 clones), the availability of full-length sequence data gives access to information on context dependence for cluster analysis, which has never been productively used before.

The combination of stringent experiment and consensus analysis shortens the time from library to improved clone (from weeks to days) compared to ribosome or phage display — first by performing fewer rounds of experimental selections and secondly by replacing the extensive biophysical analysis of individually prepared variants selected by panning methods with characterisation of just one (or a few) consensus mutants. Given that sequencing of many clones is now cheap and fast, the consensus analysis based on a BeSD output and design of a binder based on these patterns, provides a new strategy to obtain improved binders faster.

## Methods

### Display construct

The plasmid pIVEX-SNAP-HA and pIVEX anchor were constructed by Diamante^31^, based on pIVEX-SNAP-GFP vector. Plasmids pIVEX-SNAP-GFP and pIVEX-anchor are available *via* the AddGene repository. pIVEX-SNAP-GFP contains the R30I mutant of the SNAP-tag^61^. The scFv insert was cloned into pIVEX-SNAP-HA by replacing the avi-tag in front of the SNAP sequence with *Nde*I and *Kpn*I restriction sites. For the purpose of scFv selections a new vector, pISNEX, was created (for details see the Supplementary Protocol 2 and Supplementary Fig. 9A).

The benzylguanine (BG) conjugation to the pIVBT7 oligonucleotide method was adapted from the protocol published by Stein *et al*.^20^, with minor modifications (see Supplementary Protocol 3 for an updated procedure).

### Creation of the scFv error-prone libraries

The pISNEX-SNAP-HA plasmid containing the gene encoding E09 scFv or the recovered linear DNA from the second round of selection was used as a template for the error-prone PCR using the GeneMorph II random mutagenesis kit (Agilent) (see Supplementary Protocol 6 for details). A portion of the library was used for assembly into the full BeSD template and a fraction was cloned into pISNEX (by *Not*I and *Bam*HI restriction) to determine the mutation rate by sequencing a number of randomly picked colonies (74 or 45 sequences were analysed for Library I and Library II respectively).

### The assembly of the *in vitro* transcription linear DNA template

The assembly of the linear DNA template for the *in vitro* transcription and translation (IVTT) was performed essentially as described by Houlihan *et al*.^24^, with a few modifications. In brief: the assembly fragments (5′ untranslated region-AGT and 3′ HA-tag and untranslated region) were amplified in separate PCR steps with the primer pairs LMB/LMB-match and pIVBT7/pIVBT7-match (for the full list of primers used in this publication see Supplementary Table 1), respectively, from the plasmid template pISNEX-SNAP-GFP-HA. Standard thermo-cycling conditions were used with the annealing temperature of 55 °C and the 30 s extension at 72 °C (using Pfu Ultra II polymerase; Agilent). To remove the template vector the fragments were gel purified (QIAquick Gel Extraction Kit, QIAGEN), then digested with *Dpn*I (NEB), followed by ProteinaseK treatment (NEB), and finally purified with DNA Clean&Concentrator kit (Zymo Research). The 50 μl PCR assembly reaction (also with Pfu Ultra II polymerase) was done with LMB and BG-conjugated pIVBT7 primers, 20 ng of each assembly fragment and 40 ng of the insert fragment (either created with error-prone or with recovery primer pair). The standard thermo-cycling was done with annealing step at 58 °C, extension at 72 °C for 1 min 30 s and for 30 cycles. Samples were run on an agarose gel to confirm that DNA fragments with the correct size were amplified (data not shown).

### Selection by Bead Surface Display and on-bead assays

The streptavidin coated beads (5.18 μm, SiO2-MAG-SA-S1964, Microparticles) were used for the display of the SNAP-scFv-HA construct. Each selection round was performed according to the procedure described by Diamante *et al*.^31^, with the following modifications: 1) improvement of the emulsion PCR reproducibility by changing the surfactant to PicoSurf-1 (Dolomite); 2) optimisation of the IVTT conditions to accommodate for the expression of antibody fragments (e.g., lowering the expression temperature and increasing the incubation time); 3) optimising the deemulsification procedure for minimal loss of beads; 4) changing the primer pair for more efficient DNA recovery of beads selected by FACS. See the Supplementary Protocol 1 for the detailed experimental procedure.

For the on-bead assays the beads were first coated with the DNA anchors (presenting a biotin molecule on the 5′ end and BG moiety on the 3′-end), then SNAP-scFv-HA (or SNAP-HA) was expressed with PURExpress (NEB), following the manufacturers recommendations. See the Supplementary Protocols 4 and 5 for the synthesis of the anchor DNA and *in vitro* transcription/translation procedures. For the display assay the beads were incubated with 70 nM Alexa488-labeled anti-HA antibody (1 h, room temperature, shaken at 1,200 rpm), then the unbound antibody was removed by the standard wash. For the binding assay the beads were incubated (1 h, room temperature, shaken at 1,200 rpm) with 1 nM FasR-Fc, unless stated otherwise (R&D Systems), followed by an incubation (1 h, room temperature, shaken at 1,200 rpm) with DyLight488-labelled polyclonal goat anti-Fc antibody (Abcam) at 10-fold molar excess over the receptor concentration (typically 10 nM). Both steps were carried out in 1.5% marvel in PBS each was followed by three washes with PBS supplemented with Tween 20 (0.05%).

The fluorescence of the beads was analysed by flow cytometry (Cytek DxP8) and the data were analysed in FlowJo10. For the on-bead affinity analysis the Fas-Fc was titrated against the beads and the normalized median fluorescence signal values (normalised to the highest value) were fitted to the saturation binding curve equation using Prism GraphPad software (see Supplementary Fig. 3).

### Quantification of DNA molecules coupled to streptavidin beads by real-time PCR

The procedure was done as described before^31^. In brief: each PCR reaction contained 500 beads decorated with DNA templates and/or anchors were used, 0.8 μM of each primer (F-RT-1 and R-RT-1 for quantification of the template or F-RT-1 and pIVBT7 for the anchors) and 2x SensiMix SYBR No-ROX Kit (Bioline). The RT-PCR (Corbett Research Rotor-Gene 6000) program started with an initial step of 10 min at 95 °C followed by 40 cycles (95 °C for 10 s, 60 °C for 10 s, 72 °C for 5 s). Reactions were performed in duplicate and a standard curve was obtained using known concentration of linear template coding for SNAP-GFP-HA (created with unmodified LMB and pIVBT7 primers) with correlation coefficient R^2^ > 0.99. The number of DNA copies/reaction was calculated using the software accompanying the Rotor-Gene 6000 series and divided by the number of beads (500) and, in the case of DNA templates, multiplied by the correction factor 0.3 (as defined by Diamante *et al*.^31^, fraction of beads bearing DNA, according to the Poisson distribution, out of the total amount of beads).

### Phylogenetic analysis of the selection output

223 amino acid sequences of randomly selected scFvs from the fourth selection round output, were aligned using the MUSCLE algorithm^51^. Afterwards, the alignment was analysed with the Randomised Axelerated Maximum Likelihood program (RAxML) using the CIPRES online tool. The RAxML is the leading method for large-scale maximum likelihood (ML) estimation, which is a classical statistical method for phylogeny estimation. The analysis was performed using standard parameters and using bootstrap analysis (resampling method) with 100 tests. The phylogenetic tree was then visualised using the FigTree software (http://tree.bio.ed.ac.uk/software/figtree). The amino acid sequences of the scFv from each clade were aligned manually and the mutation frequency was calculated with Microsoft Excel^52^. The RAxML is the leading method for large-scale maximum likelihood (ML) estimation, which is a classical statistical method for phylogeny estimation. The analysis was performed using standard parameters and using bootstrap analysis (resampling method) with 100 tests. The resulted phylogenetic tree was then visualised using the FigTree software (http://tree.bio.ed.ac.uk/software/figtree). The amino acid sequences of the scFv from each clade were aligned manually and the mutation frequency was calculated with Microsoft Excel.

### Site-directed mutagenesis

Single-point mutants of E09 scFv as well as the R4aS and R4aH consensus scFvs were generated by saturation mutagenesis with QuikChange Lightning Site-Directed Mutagenesis Kit (Agilent) following the manufacturer’s protocol. The site-directed mutagenesis was performed on the E09 in pCantab6 vector using mutagenic primers listed in the Supplementary Table 1. The PCR amplified mutated vector was transformed in *E. coli* TG1 strain.

### Expression and characterisation of the selected antibodies and scFvs

The scFvs were expressed periplasmicaly from the vector pCantab6 in the bacterial strain TG1 and purified using nickel affinity chromatography. The IgGs were expressed and purified as described by Chodorge *et al*.^37^. The *K*_d_ values were determined by bio-layer interferometry using an Octet Red384 instrument (ForteBio, Inc.) and surface plasmon resonance (SPR) using a BIAcore T100 instrument. See Supplementary Protocols 8–10 for the experimental details.

## Additional Information

**How to cite this article**: Mankowska, S. A. *et al*. A Shorter Route to Antibody Binders *via* Quantitative *in vitro* Bead-Display Screening and Consensus Analysis. *Sci. Rep*. **6**, 36391; doi: 10.1038/srep36391 (2016).

**Publisher’s note:** Springer Nature remains neutral with regard to jurisdictional claims in published maps and institutional affiliations.

## Supplementary Material

Supplementary Information

## Figures and Tables

**Table 1:** Quantification of the binding between FasR and scFv and IgG variants.

Name	Optimisation	Mutations	**Method**	*K*_d_ [nM]^2^		*k*_on_ [10^5^ M^−1^s^−1^]^2^		*k*_off_ [10^−4^ s^−1^]^2^		*K*_d_ gain^3^	
				scFv	IgG	scFv	IgG	scFv	IgG	scFv	IgG
E09^1^	(parent)	—	**BLI**	7.60 ± 2.54	15.7 ± 3.3	2.36 ± 0.52	2.36 ± 0.81	9.56 ± 2.48	26.6 ± 3.74	—	—
			**SPR**	—	13.7 ± 0.01	—	1.74 ± 0.00	—	23.9 ± 0.03	—	—
Ep6b_B01^1^	ribosome display	*VH:* T73S; *VL:* F27aS, **Y50S**, N51D, S59P, S70T, **K95aE**	**BLI**	0.71 ± 0.27	0.23 ± 0.16	2.14 ± 0.39	4.02 ± 0.29	1.25 ± 0.66	0.59 ± 0.37	11	68.8
			**SPR**	—	0.06 ± 0.00	—	7.46 ± 0.01	—	0.44 ± 0.01	—	228
A01b	BeSD	*VH:* **S25P**, V37I, Q77R, S112P; *VL:* A14V, **Y50H**	**BLI**	2.15 ± 0.52	—	1.68 ± 0.32	—	2.72 ± 0.50	—	3.5	—
A03a	BeSD	*VH:* **S25P**, K43R, Q96R, **V102N**, G104D; *VL:* P07S, **I28T, Y50S, F55L, K95aE**	**BLI**	1.09 ± 0.09	—	2.40 ± 0.97	—	1.81 ± 0.53	—	7.0	—
A05a	BeSD	*VH:* **G08S**, **L20I**, **S25P**; *VL:* **Y50H**	**BLI**	2.89 ± 0.48	—	1.53 ± 0.2	—	3.90 ± 1.05	—	2.6	—
A09a	BeSD	*VH:* S07P, V12T, **S25P**, N32D; *VL:* **Y50H**	**BLI**	2.63 ± 0.43	—	1.47 ± 046	—	2.90 ± 1.03	—	2.9	—
A11b	BeSD	*VH:* **S25P**, S113G; *VL:* **I28T**, **Y50S**, **F55L**, **K95aE**	**BLI**	1.09 ± 0.36	—	1.30 ± 0.12	—	1.11 ± 0.18	—	7.0	—
A07a	BeSD	*VH:* **S25P**, S65G, V102N, G104D; *VL:* **Y50S**	**BLI**	0.40 ± 0.09	1.02 ± 0.24	1.62 ± 0.25	2.77 ± 1.15	0.56 ± 0.15	1.71 ± 0.20	19	15.5
			**SPR**	—	0.29 ± 0.00	—	2.30 ± 0.00	—	0.67 ± 0.01	—	47.2
R4aS	consensus design	*VH:* **S25P**, **V102N**, **G104D**; *VL:* **I28T**, **Y50S**, **F55L**, **K95aE**	**BLI**	0.38 ± 0.16	0.09 ± 0.02	1.17 ± 0.06	2.49 ± 0.69	0.45 ± 0.19	0.12 ± 0.02	20	167
			**SPR**	—	0.14 ± 0.00	—	2.82 ± 0.00	—	0.38 ± 0.01	—	97.9

Values for *k*_on_, *k*_off_ and *K*_d_ were measured by bio-layer interferometry (BLI) or surface plasmon resonance (SPR) for binding to recombinant human FasR. All measurements were performed at least in duplicate. The table presents average values with standard errors.

The consensus mutations identified by BeSD are highlighted in bold.

^1^E09 (parent) and Ep6b_—_B01 scFv were developed previously by Chodorge *et al*.^37^,^58^.

^2^Values for *k*_on_, *k*_off_ and K_*d*_ were calculated following bio-layer interferometry (BLI) or surface plasmon resonance (SPR) analysis of scFvs binding to the recombinant FasR. The binding curves were fitted according to the 1:1 Langmuir binding model using the Biacore T100 Evaluation or FortebBIO data processing software. See the Materials and Methods section for the experimental details.

^3^The *K*_d_ gain was calculated as the ratio of the parent E09 to the *K*_d_ of the variant (calculated with the raw numbers).
