# Supplementary material for: A Shorter Route to Antibody Binders via Quantitative in vitro Bead-Display Screening and Consensus Analysis
Source: Sci Rep. 2016 Nov 7;6:36391. doi: 10.1038/srep36391 (PMC5098251; doi:10.1038/srep36391)
Supplement: Supplementary Information [file srep36391-s1.pdf]

Supplementary Information for A Shorter Route to Antibody Binders *via* Quantitative *in vitro* Bead-display Screening and Consensus Analysis

Sylwia A. Mankowska[1,2], Pietro Gatti-Lafranconi[1], Matthieu Chodorge[2], Sridharan Sudharsan[2], Ralph R. Minter[2] and Florian Hollfelder[1]*.

[1] Department of Biochemistry, University of Cambridge, 80 Tennis Court Road, Cambridge CB2 1GA, UK.
[2] Antibody Discovery and Protein Engineering, MedImmune Ltd, Milstein Building, Granta Park, Cambridge, CB21 6GH, UK.

*To whom correspondence should be addressed. E-mail: fh111@cam.ac.uk

Table of Contents

Supplementary Protocols

1. Protocol for an scFv evolution cycle using BeSD. 3
2. Creation of the pISNEX plasmid. 5
3. Updated procedure for preparation of the benzylguanine modified primer. 5
4. Preparation of the spiking anchors. 6
5. *In vitro* expression of the SNAP-scFv-HA fusion for on-bead assays. 6
6. Error-prone library generation. 7
7. Delfia immunoassay for binding validation on supernatant-leaked scFvs. 7
8. Expression of scFv and IgG1 antibodies. 8
9. Bio-layer interferometry (OCTET). 8
10. Binding kinetic and affinity measurement by BIAcore analysis. 9

Supplementary Figures

Figure 1. A correlation between the number of displayed scFv molecules and antigen binding fluorescence signal. 10
Figure 2. Improvement of the *in vitro* expression and the BeSD construct for scFv display. 11
Figure 3. Antigen titration curve for an on-bead binding assay to determine K_d. 12
Figure 4. On-bead K_d measurement. 13
Figure 5. Delfia immunoassay screening of supernatant-leaked scFvs. 14
Figure 6. Sequence analysis of the V_H and V_L of the scFvs from the BeSD screening output. 15
Figure 7. Contributions of the individual consensus mutations to the affinity improvements. 16
Figure 8. Structural modelling of the scFv mutants. 17
Figure 9. Map of the pISNEX vector and IVTT DNA template. 19
Table 1. Primer list. 20

References 21

Supplementary Protocols

Supplementary Protocol 1.
Protocol for an scFv evolution cycle using BeSD.

The following procedure was optimised for selection of scFv fragments. The following steps refer to Fig. 1. The most important modifications of the procedure include: determining the most appropriate temperature for the *in vitro* translation (25 °C, Supplementary Fig. 2A), addition of a disulfide bond enhancer (leading to 2-fold increase in expression and binding signals, data not shown) as well as finding the optimal orientation of the SNAP fusion (C-terminal preferred over N-terminal fusion; see Supplementary Fig. 2B-C).

Step 1— Preparation of the emulsion PCR (ePCR) reaction. The ePCR was performed with Titanium polymerase (Clonetech), which showed the highest efficiency in ePCR compared to all other tested polymerases[1]. The standard PCR reaction mix was prepared as follows (total reaction volume of 18 µl): 1x Titanium buffer, 5′-modified biotin-forward primer (BB-LMB) and 5′ BG-modified reverse primer (pIVBT7-BG) at 0.2 µM each, 1.7 x 10^7 copies of DNA template and ~10^6 streptavidin-coated beads.

The aqueous phase was mixed with 100 µl of an oil phase. The oil phase was composed of the fluorinated surfactant (PicoSurf-1, Dolomite) as a 2.5% (w/w) solution in the oil HFE7500 ($n\text{-}C_3F_7CF(OC_2H_5)CF(CF_3)_2$, 3M NOVEC). The emulsion was created by vortexing aqueous and oil phase in PCR tubes for 3 min (at ¾ of the maximal vortex speed). Then the excess of 70 µl of the oil was removed from the bottom of the tube (in order to lower the volume of the mixture, to bury it sufficiently deep in a thermocycler's heating block).

Step 2 — Temperature cycling. The ePCR temperature program started with a ramp from 25 °C to 94 °C (1 °C/s), followed by 2 min at 94 °C and 30 cycles of denaturation (94 °C C, 30 s), annealing (48 °C, 30 s) and extension (72 °C, 1 min 30 sec for the scFv construct). After a final extension step (72 °C, 5 min), samples were incubated first at 45 °C (5 min) and then at 25 °C (20 min) to allow the biotinylated PCR products to attach to the beads.

Step 3 — De-emulsification. HFE7500 emulsions were broken by adding PBS with 0.05% Tween20 (PBS-T; 200 µl, to increase the volume of the aqueous phase for easier handling and disruption of the oil/water interface with Tween) followed by addition of 20 µl of 1H,1H,2H,2H-perfluorooctanol (PFO, Alfa Aesar). Then, the tube was gently inverted 10 times

to break the emulsion. The upper, aqueous phase was transferred to a clean Eppendorf tube containing 500 µl of PBS-T (to dilute the carried over PFO). The beads were washed twice (using a magnet to retain the beads) with deionized water and resuspended in 30 µl of deionized water.

Step 4 — Addition of the spiking anchors. A specific concentration of the anchor DNA (usually 10^7 anchor molecules/bead) was incubated with the beads in the binding buffer (5 mM Tris/HCl, 0.5 mM EDTA, 1 M NaCl, pH 7.5) at room temperature for 30 min with shaking. The non-immobilized spiking anchors were removed by washing the beads twice with water. The number of copies of PCR products and anchors per bead was quantified by real-time PCR (RT-PCR) using primers F-RT-1 and R-RT-1 or F-RT-1 and pIVBT7, respectively.

Step 5 and 6 — In vitro expression in emulsion droplets. *In vitro* transcription and translation (IVTT) reactions were carried out using the PURExpress (*In Vitro* Protein Synthesis Kit, NEB). Reactions of 25 µl (in an 1.5 ml Eppendorf tube) contained 10 µl of component A, 7.5 µl component B, 1 µl of each disulphide bond enhancer kit component (NEB) and 0.5 µl of RNAse inhibitor (NEB). The volume was adjusted with nuclease-free water (Ambion). The reaction mix was added to the beads and emulsified as in the Step 2, with the difference that the oil contained 0.5% of the surfactant. The samples were incubated at 25 °C for 4-5h.

*Step 7 — De-emulsification***.** The oil phase was removed, and then PBS-T (500 µl) was added followed by PFO (50 µl). The tube was inverted gently to allow phase extraction of the beads into the PBS-T. Subsequently the beads were washed once with PBS-T and twice with PBS (500 µl each). The beads were re-suspended in 50 µl of water. A subset of the beads was removed to perform a display assay (see Fig. 2A and Materials and Methods) to verify the percentage of beads displaying the scFv fusion (in order to determine the number of screened beads).

Step 8 and 9 — Detection of the on-bead binding. The beads were incubated sequentially with 1 nM FasR-Fc then with 10 nM of anti-Fc DyLight488-labeled antibody. For a detailed procedure describing the on-bead binding assay see Materials and Methods.

Step 10 — Fluorescence-activated sorting. Fluorescence-activated sorting was performed with a Beckman Coulter MoFlo MLS high-speed cell sorter. Beads with fluorescence above

a chosen fluorescence value (typically 0.5% of the population) were sorted in 96-well PCR plates (300 beads/well) containing 20 µl of nuclease-free water.

Step 11 — Recovery PCR. The sorted beads were used as templates in a PCR reaction with Titanium polymerase with the PCR recovery primer pair (see Supplementary Table 1). The PCR mix was prepared according to the manufacturer's guidance, and the cycling conditions were as follows: 35 cycles of denaturation at 95 °C for 30 s, annealing at 58 °C for 30 s and extension at 72 °C for 2 min, with the final extension for 5 min at 72 °C. Then, the amplified fragment was assembled into a full BeSD DNA template for the next selection cycle and also cloned into pISNEX and transformed into TOP10 cells. 88-264 transformants were picked and grew in 96-well plates, then a part of the cultures was analysed by sequencing and the other part stored at -80 °C as a glycerol master plate.

Supplementary Protocol 2.
Creation of the pISNEX plasmid.

This plasmid was derived from pIVEX-SNAP-GFP [1]. A synthetic gene was designed to replace the previous GFP construct and ordered from GeneScript. The modifications allowed cloning a protein of interest between SNAP and HA-tag using *Not*I and *Bam*HI restriction sites (for the construct map see Supplementary Fig. 9B). Additionally, the avi-tag and thrombin digestion sites were removed from the construct. Finally, the HA-tag had two flanking *Spe*I restriction sites introduced, allowing easy one-step removal of the tag if required. For this work three constructs were prepared – pISNEX-SNAP-HA (without an insert) and pISNEX-SNAP-HA with either a GFP or an scFv insert.

Supplementary Protocol 3.
Updated procedure for preparation of the benzylguanine modified primer.

The lyophilised, 5′-thiol-modified pIVBT7 oligonucleotides (~500 µg, Sigma) were resuspended in 300 µl of the deprotection buffer (100 mM Tris-HCl, pH 8.5 and 100 mM DTT) and incubated for 1 h at room temperature. Excess DTT was removed by gel filtration (NAP-5 columns, GE Healthcare) using PBS as running buffer (primer eluted in 700 µl). Subsequently O^6-benzylguanine-maleimide (BG-maleimide; 2 mg, NEB) was resuspended in 300 µl of DMF (Sigma), added to the primers and incubated for 2 h at 40 °C. BG-maleimide-

labeled oligonucleotides were purified by gel filtration, using two NAP-5 columns (GE Healthcare), each of which processed 500 μl of the primer and nuclease-free water (Ambion) as a solvent. The BG-maleimide labeled oligonucleotides eluted in 750 μl and the typical final concentration of the modified primer obtained was 97-143 ng/μl (which for the primer pIVBT7 corresponds to 10 μM).

Supplementary Protocol 4.
Preparation of the spiking anchors.

Anchors were created by PCR with TAQ polymerase (Bioline) using the pIVEX-anchor vector as template and following the manufacturer's recommendations. The anchors were prepared with bis-biotinylated (BB-) LMB forward primer and reverse pIVBT7 primer either with BG-modification (or without, in the case of negative controls). The standard cycling program was run with annealing step at 55 °C and 40 s elongation step at 72 °C. The PCR product was purified (Zymogen PCR purification kit, Zymo Research) and subsequently the desired number of spiking anchors was incubated with beads.

Supplementary Protocol 5.
In vitro expression of the SNAP-scFv-HA fusion for on-bead assays.

Streptavidin coated beads (5.18 μm, SiO_2-MAG-SA-S1964, Microparticles) were washed using a magnetic separator (DynaMag-2 Magnet, Invitrogen) to remove the storage buffer: 3 times with 1 ml of PBS supplemented with Tween 20 (0.05%), 3 times with 1 ml PBS and 3 times with 1 ml nuclease-free water. Subsequently the beads were coated with anchors analogously to step 4 of the BeSD Supplementary Protocol 1. Then, SNAP-scFv-HA (or SNAP-HA) was expressed with PURExpress (NEB), following the manufacturers recommendations. In brief, 25 μl reaction mix contained 10 μl of component A, 7.5 μl component B, 250 ng of the plasmid, 1 μl of each component of the disulphide bond enhancer kit (NEB; enhancers were not added when SNAP-HA was expressed) and 0.5 μl of RNAse inhibitors (NEB), and then the volume was adjusted with nuclease-free water (Ambion). The reaction mix was added to the anchor-coupled beads, then SNAP-scFv-HA or SNAP-HA construct was expressed for 4 h at 25 °C or 37 °C, respectively (unless otherwise stated in the

text). The unbound SNAP-fusion was removed by washing the beads with standard washing step - once with PBS containing 0.05% Tween20, then twice with PBS.

Supplementary Protocol 6.
Error-prone library generation.

Conditions for a low mutation rate epPCR (0-4 mutations per gene) were utilised according to the manufacturer's recommendations using primers F_epPCR_pisnex and R_epPCR_pisnex (see Supplementary Table 2). Briefly, a 50 µl reaction contained 3.12 µg template plasmid DNA or 751 ng of linear template (the amount of input DNA was calculated in a way to supply to the reaction ~600 ng of the 842 bp template gene), 1x Mutazyme II reaction buffer, dNTPs at 200 µM, forward and reverse primers at 0.3 µM each, and 2.5 units Mutazyme II DNA polymerase. Thermo-cycling consisted of an initial heat activation step for 2 min at 95 °C followed by 30 cycles of denaturation at 95 °C for 30 sec, annealing at 60 °C for 30 s and extension at 72°C for 1 min 30 s followed by a final elongation step at 72 °C for 10 min. The PCR product was digested with *Dpn*I (NEB) to digest any remaining plasmid and subsequently run on 1% agarose gel, extracted and dialysed against water on 13 mm mixed cellulose membrane filters (Millipore) for maximal purity.

Supplementary Protocol 7.
Delfia immunoassay for binding validation on supernatant-leaked scFvs.

Screening assays were essentially performed as described[2,3]. In brief: supernatant-leaked scFvs were immobilised (2 h) onto high binding COSTAR plates (Corning, NY, USA). Binding of human FasR-Fc fusion protein (R&D Systems) incubated (2 h) in blocked plates (1 h, 3% skimmed milk in PBS) at 0.35 µg/ml was detected (1 h) using 0.5 µg/ml of europium-labelled anti-human IgG (Perkin Elmer). The enhancing solution was incubated with the scFvs for 30 min. All the steps were done at room temperature with the plate shaking at 300 rpm. Fluorescence signals detected at 340 nm excitation and 615 nm emission were normalised by the cell culture density (measured at 600 nm) to take into account difference in cell growth. The E09 scFv was used as the control.

Supplementary Protocol 8.
Expression of scFv and IgG1 antibodies.

For the periplasmic expression of soluble scFv the clones in pCantab6 vector were transformed and expressed in the bacterial strain, TG1. Overnight cultures were added to 400 ml 2xTYAG media (0.1% glucose; 100 μg/ml ampicillin) and incubated at 30 °C for 2 ½ hours whilst shaking at 300 rpm (or until an OD_{600} of 0.6 was reached). Protein expression was induced with 1 mM IPTG and cells were grown at 30 °C for another 3 h. Then, the cultures were centrifuged at 6,000 rpm for 10 min at 4 °C. Subsequently the cell pellet was resuspended with 10 ml of cold TES (200 mM Tris-HCl, 0.5 mM EDTA, 0.5 M sucrose, pH 8.0), then 15 ml of cold 1:5 TES were added, mixed well and incubated on ice for 30 minutes. Finally, the cell debris was pelleted by centrifugation in a benchtop centrifuge (4,600 rpm; 30 minutes; 4 °C), and the periplasmic extract was purified on nickel sepharose (GE Healthcare) packed columns and buffer exchanged into PBS (NAP-10 columns, GE Healthcare).

For IgG expression, the V_H and V_L chains of selected antibodies were cloned into human IgG1 expression vectors as described in Persic *et al.*[4], except that an oriP fragment was included in the vectors to facilitate use with human embryonic kidney Epstein–Barr virus-encoded nuclear antigen-293 (HEK EBNA-293) cells and to allow episomal replication. Co-transfection of the heavy chain vector pEU15.1 and lambda light chain vector pEU4.4 into HEK EBNA-293 allowed whole IgG to be expressed and purified by protein A affinity chromatography (GE Healthcare, Little Chalfont, UK).

The scFv and antibody concentrations were determined spectrophotometrically using a calculated extinction coefficient based on the amino-acid sequence of the scFv or the antibody[5].

Supplementary Protocol 9.
Bio-layer interferometry (OCTET).

K_d values were determined by bio-layer interferometry using an Octet Red instrument (ForteBio, Inc.). Biotinylated soluble human FasR (Peprotech) at 5 μg/ml in 1x kinetics buffer (PBS, pH 7.4, 0.01% bovine serum albumin and 0.01% Tween 20) was loaded onto streptavidin-coated biosensors (SA biosensors; ForteBio) and incubated with scFvs. A titration of seven different scFv concentrations was used to measure kinetics with the highest concentration starting from 125 nM. Each measurement consisted of five steps: baseline

acquisition, 60 s; FasR loading onto SA sensor, 300 s; baseline acquisition, 60 s; association of scFv/IgG, 600 s; dissociation of scFv/IgG, 600 s. Baseline and dissociation steps were performed in kinetics buffer. All steps were performed with sample agitation at 1,000 rpm. Binding kinetics constants (K_d, k_{on} and k_{off}) were determined using a 1:1 Langmuir-binding model in kinetics data analysis mode using data processing software (ForteBIO). The affinity of anti-FasR IgGs was measured as above; with the difference that the analysed antibody (used at 5 µg/ml in the kinetics buffer) was immobilised on anti-human IgG Fc capture sensors (AHC; ForteBIO) and the monomeric Fas receptor was used as the ligand at seven different concentrations (with the highest concentration starting at 250 nM).

Comment: Based on our experience we consider the BLI measurements merely a useful guide to pick the better binders rather than a trusted K_d value. SPR measurements (see below) showed much lower variation when repeated (as well as typically lower standard deviation). Therefore K_d values determined by SPR were used for the final comparison between the variants.

Supplementary Protocol 10.
Binding kinetic and affinity measurement by BIAcore analysis.

The affinity and kinetic parameters of anti-FasR antibodies for the FasR were determined by surface plasmon resonance (SPR) using a BIAcore T100 instrument set up at 25 ºC. IgG analysis was performed by first capturing ~100 resonance units (RUs) of the antibody on the protein G′–C1 (planar) sensor chip (30 s capture at 5 µl/min) and running a serial dilution from 497 to 15.5 nM of FasR (Peprotech) as analyte for 3 min association followed by a 10 min dissociation phase (buffer was run at 50 µl/min). Curve fitting was done with the Biacore T100 Evaluation software 2.0.3. (Biacore) using a 1:1 Langmuir binding model and with the initial response (RI) constrained to 0.

Supplementary Figures

Supplementary Figure 1. *A correlation between the number of displayed scFv molecules and the*

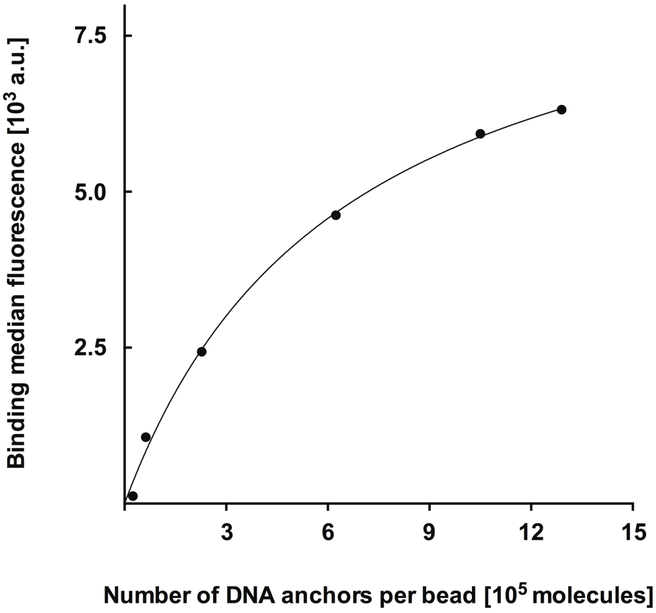

antigen binding fluorescence signal.

The number of spiking anchors was quantified by RT-PCR and can be directly translated to number of scFv molecules displayed on the bead surface. The number of coupled spacers increased the median fluorescence value of the binding to the FasR-Fc (53-fold signal increase from 120 to 6,316 a.u.). The deviation from linearity above 6 $x10^5$ anchors suggests that the beads start to become saturated with spiking anchors and that the saturation curve reaches a plateau around 1.3 x 10^6 anchors per bead. The data were fitted to a saturation binding curve using Prism GraphPad software ($Y = B_{max}*X/(K_d+X)$, where X is the number of anchors, Y is the binding fluorescence signal, B_{max} is the maximal binding response, K_d is the number of anchors to reach half-maximal binding; R^2 = 0.997).

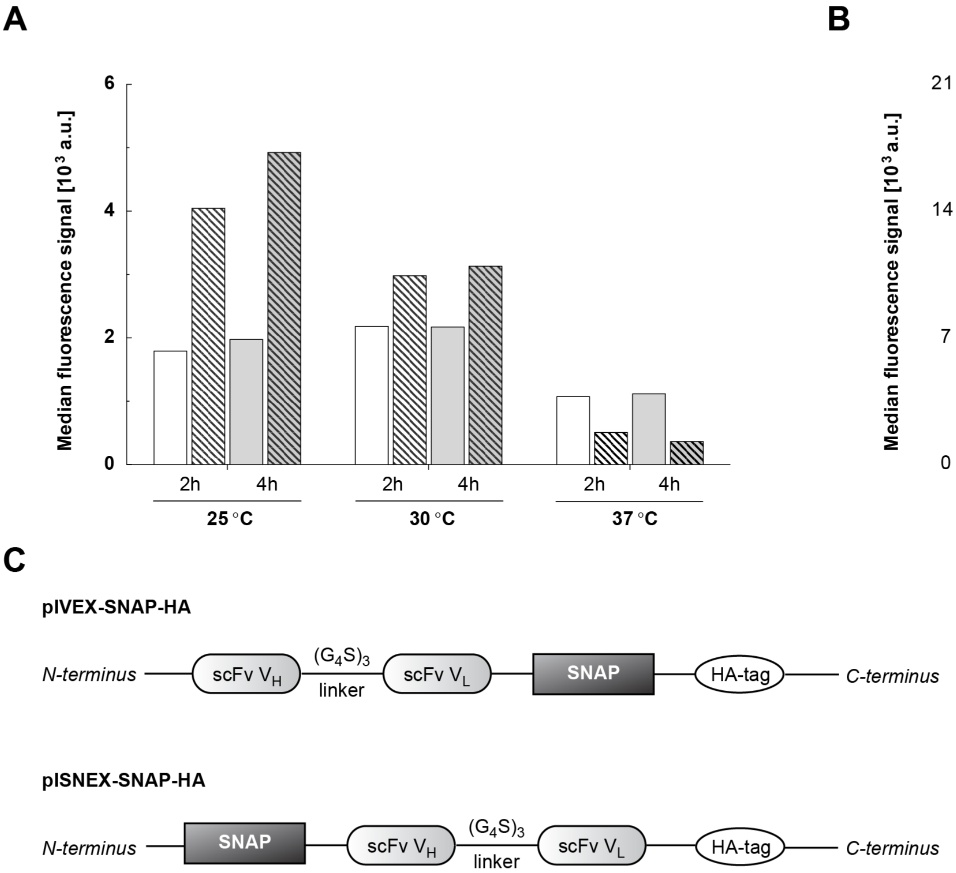

Supplementary Figure 2. *Improvement of in vitro expression and the BeSD construct for scFv display.* (**A**) Fluorescence values detected with anti-HA (plain colours) and anti-FasR-Fc (patterned bars) antibodies over a range of IVTT expression temperatures and times (2h clear bars, 4h grey bars). The scFv was expressed from pIVEX vector, and the on-bead binding assay was done at 10 nM Fas-Fc. Although absolute expression levels peaks at 30 °C, the ratio between expression and binding indicate that expression at 25 °C yields a protein with better binding ability. (**B**) On-bead display and binding assays showed 8- and 66-fold increases in median fluorescence signal (detected by anti-HA or anti-Fc antibody, respectively) for the scFv cloned on the C-terminal side of SNAP over the signal the N-terminus fusion to the SNAP. These data suggest that the fusion of the N-terminus of the scFv with the C-terminus of the SNAP results in ~8-fold better display of functional scFv than the fusion of the proteins in the reverse order. The binding assay was performed at 1 nM Fas-Fc. The on-bead assay measurements were performed in triplicate for pIVEX and in duplicate for pISNEX. (**C**) The cartoon represents the scFv protein expressed from pIVEX-SNAP-HA and pISNEX-SNAP-HA plasmids.

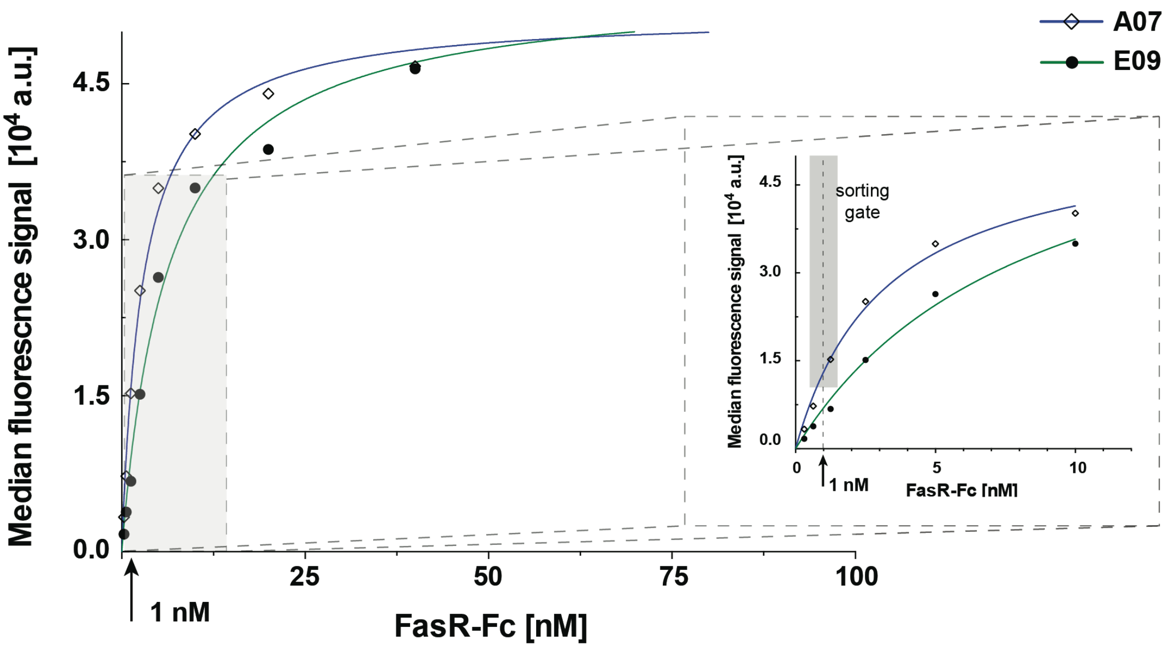

Supplementary Figure 3. *Antigen titration curve for an on-bead binding assay to determine K_d.* The binding profiles of the improved A07 scFv (blue) and the parent E09 scFv (green) were compared. The use of FACS as the screening technique allows quantitative affinity discrimination and effectively successful selection of improved binders by selection of stringent sorting gate, as attempted in selection rounds II-IV (see text and Fig. 3A). The data were fitted to the saturation binding curve using Prism GraphPad software ($Y = B_{max}*X/(K_d+X)$, where X is the concentration of the FasR-Fc, Y is the binding fluorescence signal, B_{max} is the maximal binding response, K_d is the concentration of the receptor required to reach half-maximal binding; for both curves $R^2 = 0.99$).

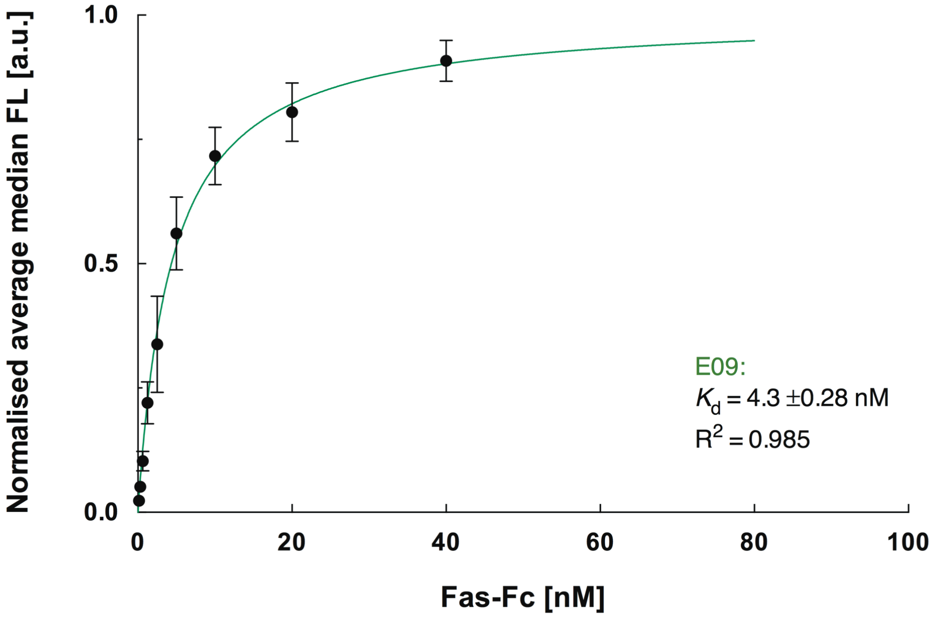

Supplementary Figure 4. *On-bead K_d measurement.*

The affinity was measured by performing an on-bead binding assay with the 10-point titration of the FasR-Fc. The plot shows the binding curve for the scFv E09. A saturation binding curve (as in Supplementary Figure 3) was fitted to the mean values of two independent experiments (average standard deviation: 13%; individual SD are shown as error bars), normalised to the maximum fluorescence obtained.

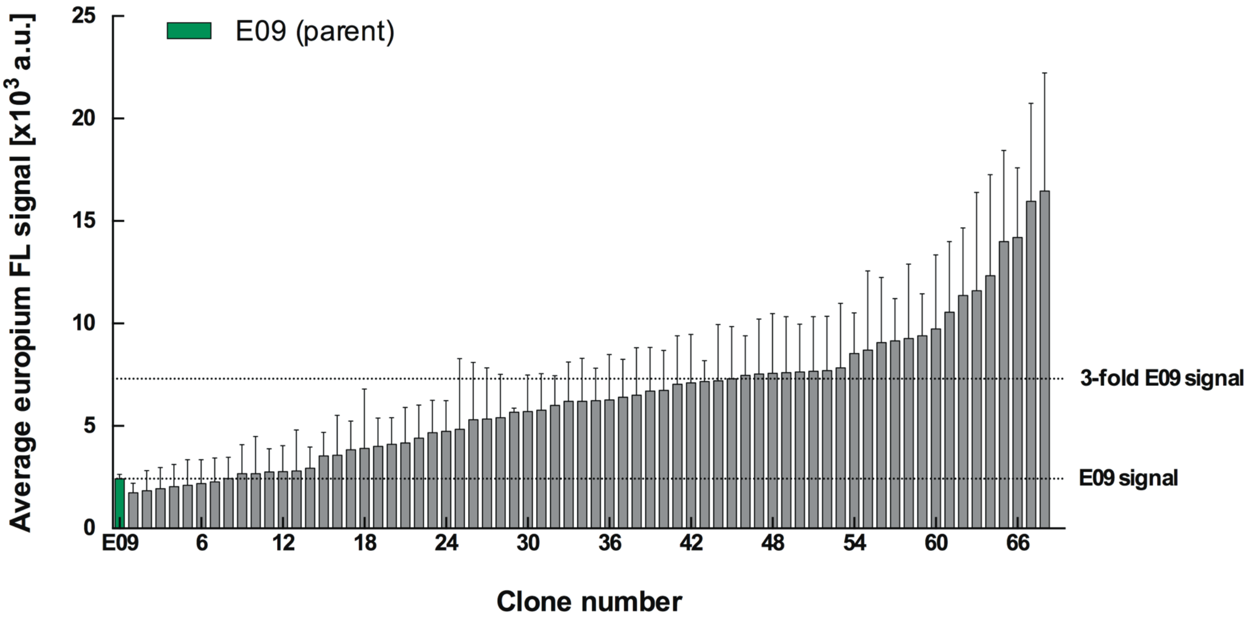

Supplementary Figure 5. *Delfia immunoassay screening of supernatant-leaked scFvs.* 93% of the screened mutants from the output of the fourth selection round showed a binding signal equal or higher than antibody E09 (lower dotted bar). Of these 38% had at least a 3-fold higher signal (i.e. fell above the upper dotted line), and thus were considered to be the significantly improved binders.

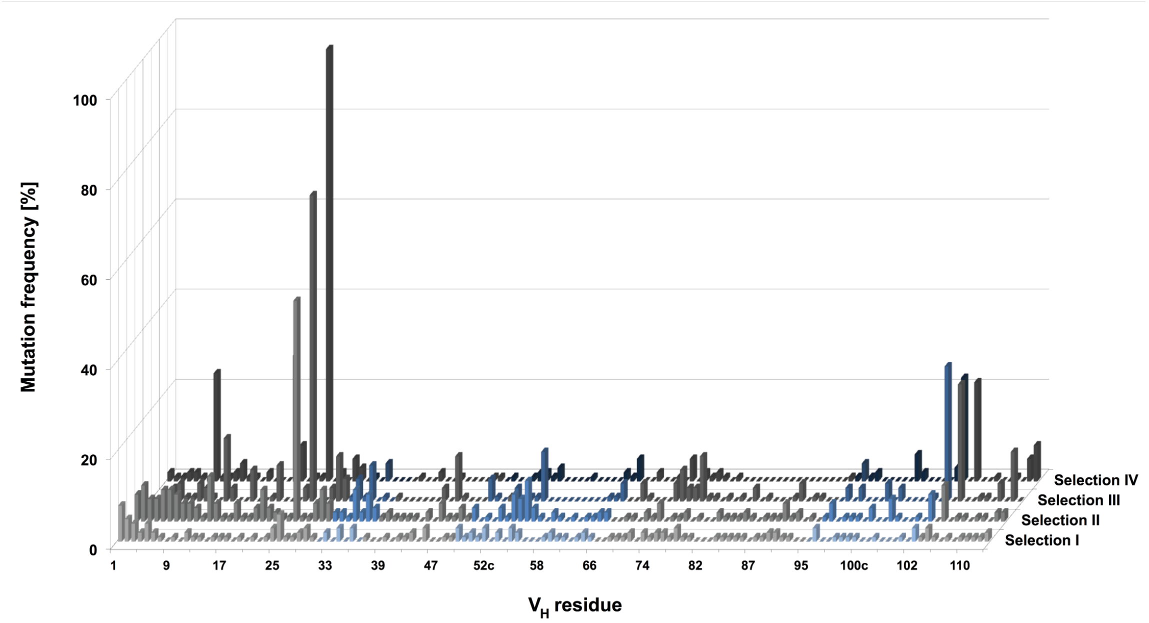

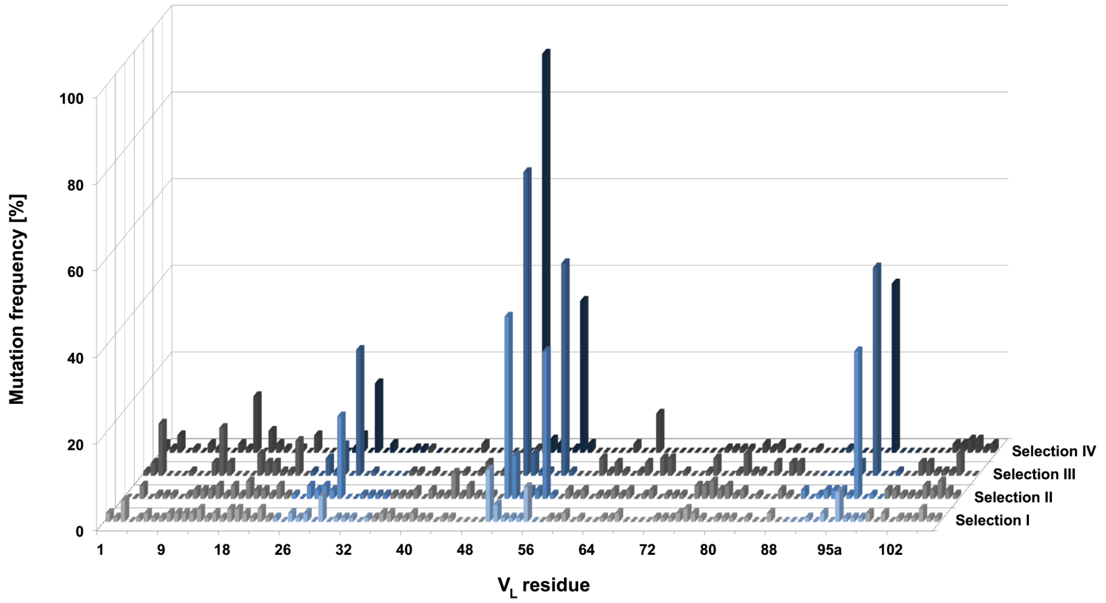

Supplementary Figure 6. *Sequence analysis of the V_H and V_L of the scFvs from the BeSD screening output.*

The scFv residues were numbered according to the Kabat numbering scheme[6]. CDR regions residues are coloured in blue and framework positions in grey. Eight hotspots[3, 7] (assigned based on the mutation frequencies in the output from the fourth selection round, see Fig. 4) were identified in positions V_H 8, 20, 25, 102, 104, V_L 50, 55 and 95a. Enrichments in these positions were observed during the progression of the selection (selection rounds I-IV). Each of these residues was preferably mutated to a specific amino acid, which suggests that those mutations were beneficial for scFv's biophysical properties. To investigate the influence of each mutation on FasR binding closer, single point mutants were created and tested by BLI (see Supplementary Fig. 7).

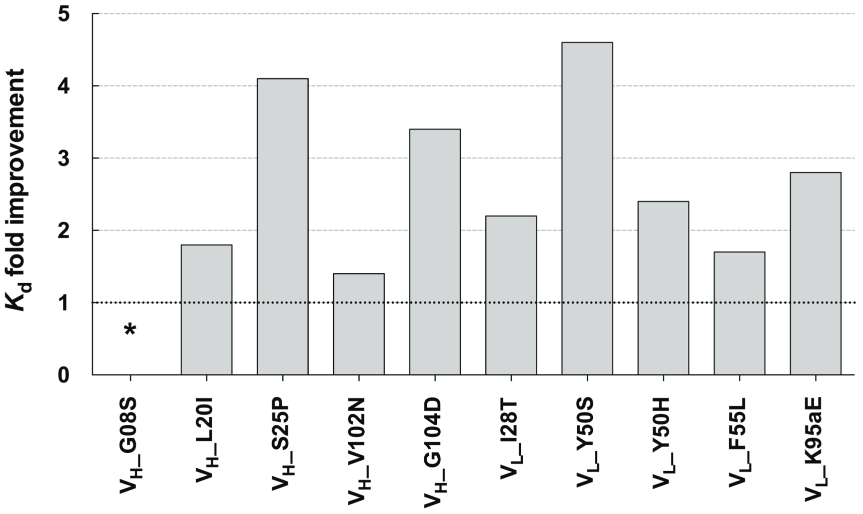

Supplementary Figure 7. *Contributions of the individual consensus mutations to the affinity improvements.*

The ten hotspot mutations identified by analysis of the amino acid sequences of the scFv population from the fourth round selection output (see Fig.5 and Supplementary Fig. 6) were individually introduced in the parent E09 to measure their impact on the affinity improvement. The affinity for recombinant FasR of the mutants and the parent E09 were determined by BLI. The graph depicts the K_d improvement (average values of minimum of 3 measurements were compared) of each variant relative to the parent antibody E09. One of the mutants (V_H G08S, marked with '*') could not be expressed in *E.coli*, and so the K_d could not be determined.

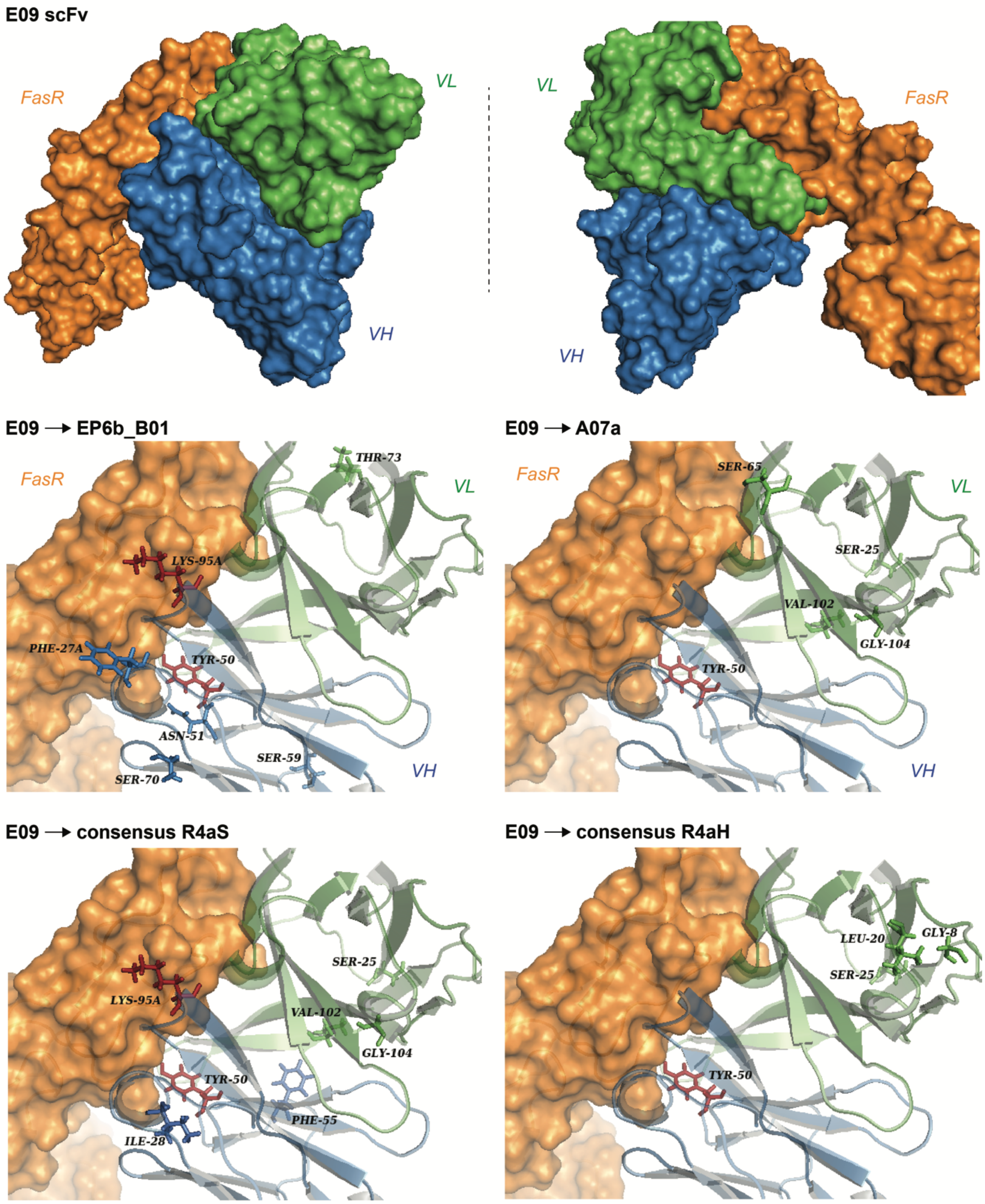

Supplementary Figure 8. *Structural modelling of the scFv mutants*....................................... The mutations present in affinity matured scFv variant isolated by ribosome display (E6b_B01), BeSD (A07a) and the consensus mutants (R4aS and R4aH) were modelled (using PyMOL software) on the structure of the E09 scFv in complex with the FasR derived by Chodorge *et al.*[3] (accessible from the Protein Data Bank with accession number 3TJE). The top panel shows the complex (mirrored image) of the heavy (green) and light (blue) variable chains of the E09 scFv with FasR (orange). The middle

and bottom panels compare the locations of the residues mutated in Ep6b_B01[3] scFv, A07b and the consensus mutants R4aS and R4aH. The epitope contact residues were highlighted in dark red and the buried residues in colours corresponding to heavy and light chains (green and blue, respectively). Four of the BeSD-selected hotspots were located in the complementarity determining regions (CDRs) of the antibody – V_H V102N, V_L Y50S, V_L F55L and V_L K95aE, but only two of them, Y50S and K95aE in the light chain, were directly involved in epitope binding. Mutations Y50S and K95aE found in both A07a and R4aS consensus scFv were also characterised previously by Chodorge *et al.*[3] in the Ep6b_B01 scFv, as residues participating in epitope binding. Indeed, V_L Y50S and V_L K95aE mutations improved the K_d by 4- and 2–fold over the parent E09, respectively (Supplementary Fig. 7). Two residues (V102 and G104) at the terminal region of the heavy chain (positioned at the interface of the heavy and light chains) were identified that were mutated in 20% of sequences. This region of the scFv is thought to have an influence on V_H–V_L domain orientation affecting the overall scFv conformation[8]. The other influential point mutation was V_H S25P mutation, which occurred in 96% of analysed sequences from the selection IV output (Fig. 4 and 5) and caused 4-fold the K_d improvement over the E09 scFv (Supplementary Fig. S7). The V_H S25P mutation occurred in a close proximity to other two mutations mentioned earlier (V_H V102N and V_H G104D) at the interface between heavy and the light chain, so it is likely that those three positions substantially affected the structure of the scFv's binding interface. The A07a scFv that had five point mutations included four that were identified as consensus mutations (V_H S25P, V_H V102N, V_H G104D, V_L Y50S) and an additional V_H mutation in CDR2 – S65G, which is not at the binding interface.

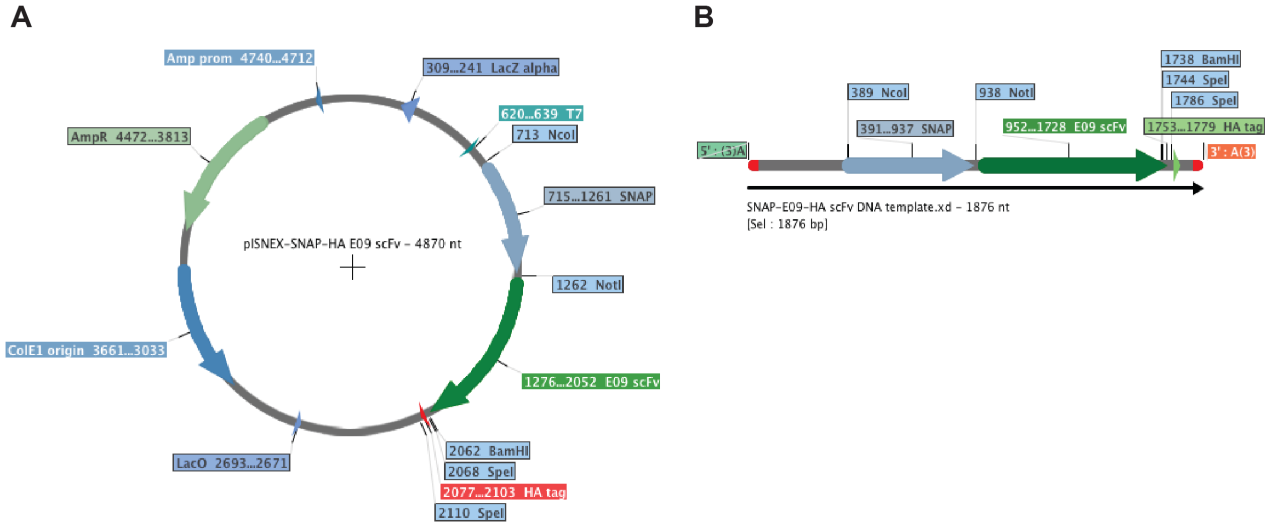

Supplementary Figure 9. *Map of the pISNEX vector and IVTT DNA template.* (**A**) pISNEX vector map. The BeSD *in vitro* expression vector was derived from pIVEX-SNAP-GFP vector. The scFv insert was cloned with *Not*I and *Bam*HI restriction sites. (**B**) BeSD DNA template map. The cloning sites are highlighted in blue. The vector and the template were visualised with SerialCloner2-6 software.

Supplementary Table 1

The list of primers used in the presented work. All oligonucleotides were ordered from Sigma at HPLC grade, except from real-time PCR and cloning primers, which were reverse-phase purified.

Primer name	Use	Sequence 5′ to 3′
(BB-) LMB	IVTT template assembly	ATGTGCTGCAAGGCGATTAAG
(BG-) pIVBT7	IVTT template assembly	AGGGGTTATGCTAGTTATTGCTCAGCGGTG
F-RT-1	(Fwd) real-time PCR	CGGCGTAGAGGATCGAGA
R-RT-1	(Rev) real-time PCR	CTAGAGGGAAACCGTTGTGG
F-epPCR-pisnex	error-prone PCT	GGCTTGGGAAGCGGCCGC
R-epPCR-pisnex	error-prone PCT	GGTACATCATACGGATAACCACTAGTGGATCC
LMB-match	assembly fragment	GCGGCCGCTTCCCAAGCC
pIVBT7-match	assembly fragment	CACTAGTGGTTATCCGTATGATGTACC
F-insert	recovery PCR	GATTTGGATGTGGGCGGTTAC
R-insert	recovery PCR	GGCTTGCATAATCTGGTACATC
F-POI	sequencing (pISNEX)	GTTGGGGAAGCCAGGCTTG
R-POI	sequencing (pISNEX)	CTTTGTTAGCAGCCGGATCTG
Lseq	sequencing (pCantab6)	GATTACGCCAAGCTTTGGAGC
myc	sequencing (pCantab6)	CTCTTCTGAGATGAGTTTTTG

References

1. Diamante L, Gatti-Lafranconi P, Schaerli Y, Hollfelder F. In vitro affinity screening of protein and peptide binders by megavalent bead surface display. *Protein Eng Des Sel* **26**, 713-724 (2013).

2. Chodorge M, Fourage L, Ravot G, Jermutus L, Minter RR. In vitro DNA recombination by L-Shuffling during ribosome display affinity maturation of an anti-Fas antibody increases the population of improved variants. *Protein Eng Des Sel* **21**, 343-351 (2008).

3. Chodorge M*, et al.* A series of Fas receptor agonist antibodies that demonstrate an inverse correlation between affinity and potency. *Cell Death Differ* **19**, 1187-1195 (2012).

4. Persic L, Roberts A, Wilton J, Cattaneo A, Bradbury A, Hoogenboom HR. An integrated vector system for the eukaryotic expression of antibodies or their fragments after selection from phage display libraries. *Gene* **187**, 9-18 (1997).

5. Edelhoch H. Spectroscopic determination of tryptophan and tyrosine in proteins. *Biochemistry* **6**, 1948-1954 (1967).

6. Kabat EA, Wu TT, Perry HM, Gottesman KS, Foeller C. *Sequences of Proteins of Immunological Interest*, 5th edn. US Department of Health and Human Services, Public Health Service, National Institutes of Health (1991).

7. Thom G*, et al.* Probing a protein-protein interaction by in vitro evolution. *Proc Natl Acad Sci U S A* **103**, 7619-7624 (2006).

8. Bujotzek A*, et al.* Prediction of VH-VL domain orientation for antibody variable domain modeling. *Proteins* **83**, 681-695 (2015).
